# Using citation networks to evaluate the impact of text length on keyword extraction

**DOI:** 10.1371/journal.pone.0294500

**Published:** 2023-11-27

**Authors:** Jorge A. V. Tohalino, Thiago C. Silva, Diego R. Amancio

**Affiliations:** 1 Institute of Mathematics and Computer Science – USP, São Carlos, SP, Brazil; 2 Universidade Católica de Brasília, Brasília, DF, Brazil; West Pomeranian University of Technology, POLAND

## Abstract

The identification of key concepts within unstructured data is of paramount importance in practical applications. Despite the abundance of proposed methods for extracting primary topics, only a few works investigated the influence of text length on the performance of keyword extraction (KE) methods. Specifically, many studies lean on abstracts and titles for content extraction from papers, leaving it uncertain whether leveraging the complete content of papers can yield consistent results. Hence, in this study, we employ a network-based approach to evaluate the concordance between keywords extracted from abstracts and those from the entire papers. Community detection methods are utilized to identify interconnected papers in citation networks. Subsequently, paper clusters are formed to identify salient terms within each cluster, employing a methodology akin to the term frequency-inverse document frequency (tf-idf) approach. Once each cluster has been endowed with its distinctive set of key terms, these selected terms are employed to serve as representative keywords at the paper level. The top-ranked words at the cluster level, which also appear in the abstract, are chosen as keywords for the paper. Our findings indicate that although various community detection methods used in KE yield similar levels of accuracy. Notably, text clustering approaches outperform all citation-based methods, while all approaches yield relatively low accuracy values. We also identified a lack of concordance between keywords extracted from the abstracts and those extracted from the corresponding full-text source. Considering that citations and text clustering yield distinct outcomes, combining them in hybrid approaches could offer improved performance.

## 1. Introduction

The abundance of textual data on the Internet has engendered a pressing demand for proficient text analysis methodologies. Online textual data encompasses diverse dimensions, both in terms of volume and typology, encompassing literary works, encyclopedic compilations and journalism. Furthermore, the exponential growth of user-generated content, including concise textual expressions, has amplified this data landscape. Examples of such user-generated content include social media messages, product descriptions, online reviews, and even scholarly research papers [1]. The imperative to condense vast information repositories has given rise to the pivotal role of keyword extraction (KE) within the domain of natural language processing (NLP). The goal of KE is to identify the most informative and relevant words or topics within a given document [2]. Keywords serve as a useful tool for users, allowing them to quickly understand the overall content of the texts. Identifying keywords is crucial because they can help condense extensive volumes of text, including lengthy documents. Furthermore, keywords play a pivotal role in enhancing search capabilities and facilitating knowledge exploration. Lastly, keywords are instrumental in suggesting supplementary content across various applications. The significance of keyword extraction becomes evident through its pivotal role in numerous NLP applications, including text categorization, document summarization, document tagging, recommendation systems, speech recognition, and many more [1, 3].

The KE task has been the subject of numerous studies. These investigations can be broadly classified into three categories: statistical methods, linguistic/syntactic approaches, and graph-based methods. Different paradigms have also been used in a combined approach for supervised classification, where the extracted features are employed in a machine learning algorithm for a binary classification task [4]. While these methods have demonstrated effectiveness in processing large texts, they present significant challenges when applied to short texts with high sparsity [5].

The identification of keywords within short texts, specifically in scientific manuscripts, poses a significant challenge, particularly when utilizing open scholarly datasets that only provide the title and abstract as sources of textual information [6]. The challenge of extracting keywords from short texts, particularly in the case of scientific papers, has motivated the development of some approaches. One proposed method for addressing the sparsity of abstracts is to group abstracts using clustering techniques [7]. This can be accomplished by utilizing citations as a proxy for determining the similarity between papers, thereby circumventing the need for direct comparison of the short texts. Despite the use of such clustering and other external information [1, 5], there is a lack of comprehensive studies comparing the compatibility of keywords extracted from abstracts and full texts. The accurate representation of the semantic information present in scientific papers is crucial in many areas, as it forms the foundation for many scientometric studies. Thus, this study aims to address the following research questions:

1. To what extent are keywords extracted from abstracts similar to those extracted from the corresponding full papers?
2. Is there consistency in the set of keywords extracted by distinct community detection methods?
3. Does using citations result in superior performance as compared to directly assessing abstract similarity via textual information?

We employed clustering methods to extract keywords from abstracts and compared them with keywords extracted from the corresponding full texts. Using a citation network, we evaluated the performance of various established community detection methods in identifying groups of related papers for the purpose of keyword extraction. We also evaluated clustering approaches that do not rely on citation information (e.g. KMeans and tf-idf), including techniques based on neural embeddings (BERT).

The study revealed several interesting results. All evaluated methods were found to have a considerable discrepancy with keywords found in the full texts. We observed that clustering methods that rely solely on textual information outperformed those based on citation networks, indicating that citations may not be an optimal proxy for semantic similarity. Furthermore, our results indicate that the various community detection strategies evaluated yielded similar performance levels, despite the observed differences in the set of keywords identified by each approach. One potential explanation for the ineffectiveness of citations as a cohesive indicator may stem from the observation that a considerable portion of citations fails to mirror content similarities, a point underscored by related literature. Consequently, the grouping of papers based on citation patterns may result in clusters of papers exhibiting disparate content, thereby potentially influencing the process of keyword identification.

In summary, our findings suggest that the quantity of information used to extract keywords can strongly impact the performance of the task. Therefore, studies using similarity networks should consider the use of full texts, when available, to provide more robust information regarding topics extracted from paper networks. Given our observation of substantial dissimilarity between information derived from full-text articles and abstracts in the context of keyword extraction, one might contemplate employing hybrid methodologies that integrate both categories of data sources. Furthermore, such a hybrid approach could also encompass the incorporation of citation data and textual content analysis for enhanced keyword extraction.

The structure of this paper is as follows. In Section, we present a comprehensive review of the most pertinent studies in the area of keyword extraction. The proposed methodology for extracting keywords from both short and long texts is outlined in Section, which also includes information regarding the adopted datasets. More specifically, Section describes both text pre-processing and network construction techniques. The methodology includes the identification of cohesive paper clusters using community detection techniques and the framework employed for deriving keywords for individual papers from the aggregate keywords extracted from paper clusters. The main results are presented and discussed in Section. Here we first investigated whether words in the abstract are present in the full text and vice versa. Subsequently, we scrutinized the concordance between keywords identified in the abstracts and those in the full texts. Finally, in Section 4.1, we summarize with conclusions and suggest potential perspectives for future research.

## 2. Related works

Traditional techniques for keyword extraction can typically be categorized into two primary classes: statistical and graph-based approaches. One prevalent constraint in many of these methods is the necessity for the text to possess a minimum length to ensure the significance of word frequencies or to prevent the extracted graph from resembling a mere linear structure. This challenge can be addressed by identifying keywords within a collection of shorter texts, rather than a single document.

Unlike other papers that aim to introduce new keyword extraction methods, our study involves a comparative analysis of established techniques (such as the ones described below) for extracting keywords from both short and lengthy texts. Our primary objective is to assess the congruence between keywords extracted from the abstracts and those derived from the complete content of research papers.

### 2.1 Statistical KE techniques

The early works that addressed the keyword extraction problem focused on statistical methods. The spatial distribution of words along the text is used to gauge words’ relevance [8]. The most simple approach is based on word frequency, where words with higher frequency values are considered keywords. However, these methods do not consider word order, therefore, if the text is shuffled, a meaningless version of the text would generate the same set of keywords. The combination of frequency and spatial distribution was then proposed to address this issue via word clustering and entropy [8, 9]. The idea behind these methods is that important words are commonly concentrated in certain parts of the text, where the main topics are located. In this sense, irrelevant words are distributed regularly along texts, while keywords present an uneven distribution and tend to form semantic groups. Another improvement to frequency-based methods is the tf-idf approach, which weights the importance of a word according to its frequency within a text and the frequency along the dataset. The main advantage of these methods is that they are simple and do not require an external corpus or knowledge of the language. [2] proposed a method for selecting keywords based on the informativeness value of each word. This score was calculated at the corpus, cluster, and document levels. At the corpus level, the informativeness was computed taking into account all the documents, while at the cluster level, the word importance was calculated within a group of related texts. The results from the previous steps were then used to compute the informativeness at the document level. This approach yielded a good performance for extracting keywords.

### 2.2 Graph-based KE techniques

While pure statistics are generally simple methods, some techniques such as tf-idf do not account for the spatial distribution of words along the text. As such,

Graph-based methods have also been used approaches to model texts [10]. Several works addressed the keyword extraction problem representing documents as word co-occurrence networks, where two words are connected if they co-occur in a given context [11]. Centrality metrics are then used to assign an importance value to each word. [12] concluded that network metrics are able to successfully extract relevant words for the keyword extraction task. They also highlighted that network-based approaches do not need the use of external corpora and they are language independent.

The use of word embeddings and large contexts has also been useful in improving the quality of co-occurrence networks when extracting keywords [11]. A different approach was proposed by [13], where community detection methods were applied to a network of semantic relationships. Community detection methods are used to cluster together related (similar) concepts that appear in the same context. The authors used Wikipedia to establish the semantic relatedness between the words of the document. According to [13], important words tend to be grouped into highly connected communities, which are related to the main topics of the document.

Several studies have enhanced TextRank [14] by incorporating different semantic relationships between words as node weights for the word ranking algorithm. For instance, [3] used Wikipedia as an external knowledge base, while [1] employed the Word2Vec and Doc2Vec embedding models to compute the semantic similarity between words.

In order to address the keyword extraction problem in short texts, several works rely on the use of semantics and background knowledge. According to [5], extracting only basic or straightforward features from the words is insufficient for finding keywords from short texts. [1] remarked that text clustering approaches could prove valuable in mitigating the semantic scarcity found in short texts. These techniques enable the clustering of related texts, thereby allowing for the extraction of more semantic information by aggregating texts in the same cluster. [15] employed clustering algorithms to identify the most relevant words for each cluster, operating under the hypothesis that texts with similar topics contain similar keywords. Then, a graph-based approach was applied to each text cluster; and the PageRank algorithm was used to extract keywords.

## 3. Material and methods

**Fig 1:**
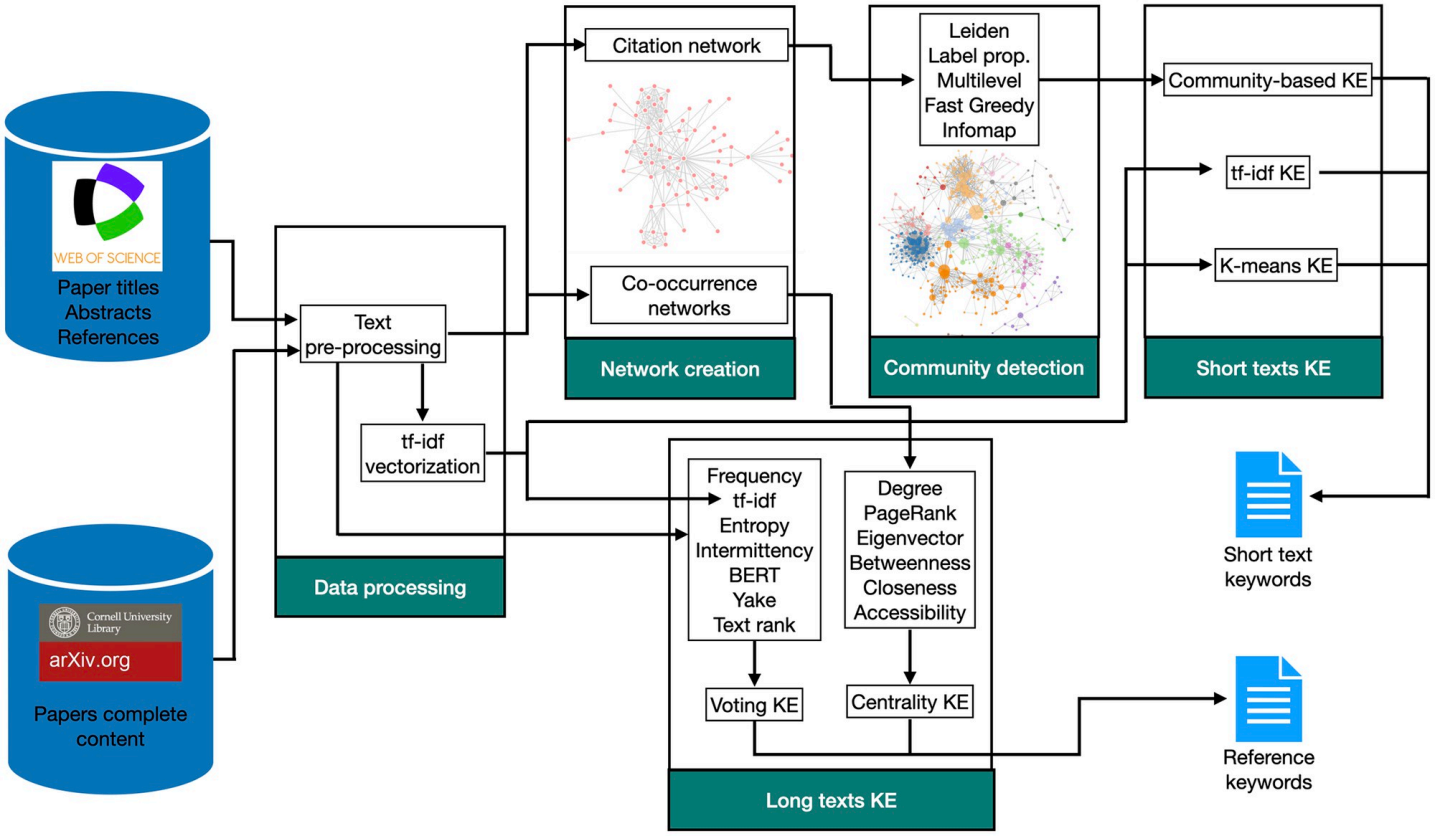
The workflow of the adopted keyword extraction system involves several stages. The initial step is pre-processing of the text. Next, a citation network is constructed and community detection algorithms are employed to extract keywords from short texts. For comparative evaluation, we implemented both a tf-idf and K-Means approach. Both approaches do not consider citations to cluster papers. In order to generate reference keywords, we employed a combination of statistical and traditional keyword extraction methods on the full texts. Additionally, we also evaluated a co-occurrence network approach as an alternative method for keyword extraction.

The framework proposed to extract keywords comprises the following main steps: i) text pre-processing; ii) network construction; iii) community detection; iv) short texts keyword extraction; and v) long texts keyword extraction. The steps are summarized below and illustrated in Fig 1. Herein, we present an overview of the adopted methodology, with each individual step detailed in the subsequent subsections.

1. *Text pre-processing*: this phase comprises the text-processing and vectorization steps. The first step includes the removal of stopwords. The remaining words are stemmed and the tf-idf approach is employed to obtain the vectorized form of the pre-processed texts. Additional details on the pre-processing steps applied can be found in Section.
2. *Network creation*: we first constructed a paper citation network, which is used for short text keyword extraction. We also modeled the complete content of each paper as word co-occurrence networks, which were used to extract keywords from long texts. In Section, we describe the required steps for the creation of both network models.
3. *Community detection*: we applied community detection methods to the citation networks in order to find clusters of related papers (see Section).
4. *Short texts keyword extraction*: this phase is responsible for the extraction of keywords from short texts (paper abstracts). The clusters obtained in the previous step are used in this phase. The relevance of each word is computed inside and outside communities. We also proposed two methods for keyword extraction based on tf-idf and the K-Means algorithm.
5. *Long texts keyword extraction*: to identify reference keywords, we used the complete content of the papers as input from several well-known keyword extraction methods. We evaluated methods based on word frequency, tf-idf, entropy, intermittency, BERT, Yake and TextRank [8, 14, 16–18]. We also used a network approach based on co-occurrence networks and centrality metrics to find keywords for long texts. These networks were characterized using centrality metrics. A detailed explanation of the adopted methodology is shown in Section.

### 3.1 Datasets

The following two datasets were used:

1. *Short texts KE dataset*: we used the dataset collected in [7]. The authors retrieved the information from 11,063 papers on the complex networks field. Our choice to use papers pertaining to the complex network domain is predicated on our status as researchers within this field. This allows us to conduct a more comprehensive examination to determine the degree of congruence between the keywords generated by various techniques and the established tenets of this domain.
  The data was obtained from the *Web of Science* (WOS) database. The selected papers were published from 1991 to 2013. For each paper, the authors extracted the title, abstract, and list of references. The latter was used to construct a citation network. The title and abstract of each paper were used as input for the application of keyword extraction techniques.
2. *Long texts KE dataset*: In order to generate a list of reference keywords for each abstract, we collected the full content of each paper, including the introduction, methodology, results and discussion, conclusions, and S1 Appendix sections of each research article. We used the API of the arXiv database to extract the complete content of the papers. We performed an automatic search using both the title and the abstract of each paper as keywords for the arXiv API.

Table 1 presents a summary of the statistical information for the datasets. The information provided was calculated from the pre-processed versions.

**Table 1:** Statistical information from datasets. |*D*| stands for the number of documents. We also show the average number of tokens (*W*_*avg*_), and the first (*W*_*q*1_) and third (*W*_*q*3_) quartiles of the distribution of the number of tokens. Similarly, *U*_*avg*_ represents the average vocabulary size, while *U*_*q*1_, and *U*_*q*3_ are the first and third quartiles of the vocabulary size distribution, respectively.

Dataset	|*D*|	*W*_*avg*_	*W*_*q*1_	*W*_*q*3_	*U*_*avg*_	*U*_*q*1_	*U*_*q*3_
Paper Abstracts	11,063	79.12	59.00	95.00	55.97	43.00	66.00
Full text	1,982	2,020.58	1,170.25	2,405.00	517.64	394.00	595.00

### 3.2 Data processing

This phase comprises three steps: data preparation, text pre-processing, and tf-idf vectorization. The data preparation step consisted of processing the recovered papers from the arXiv database (for the full content of the papers). We obtained a LaTeX version of each paper, so we had to remove all LaTeX tags. We also removed the authors list, institutions, and acknowledgments from the cleaned text. The following sections were included in the analysis of full papers: introduction, related works, methodology (or materials and methods), results, discussion, conclusion, and S1 Appendix sections.

The following procedure was used to extract data from arXiv. To access the arXiv library, we utilized Python and connected to it via the arXiv API. This API provides a handy ‘arxiv.Search’ function that allowed us to input the title and abstract from the Short Texts dataset as parameters. The API, in response, retrieved the most relevant results closely matching our query, which combined both the title and abstract of the papers. We then implemented the bag-of-word cosine similarity metric to compare our query against each of the titles and abstracts retrieved by the API. If the similarity score exceeded a set threshold, which was typically 0.9 or higher, we chose the most closely matching data. It’s important to note that we did not insist on an exact match (a similarity score of 1) since some authors might later modify their titles or abstracts on arXiv. For those results that did not meet the similarity threshold, we disregarded them. In cases where the search was successful, we obtained the corresponding link. Within these links, we located the .tex and .pdf files containing the complete content of the papers. Subsequently, we retrieved both the .pdf and .tex files and processed them to extract plain text from each paper. This systematic procedure enabled us to access the entire content of these articles.

Text pre-processing transformations were applied to all texts of the dataset. We first removed stopwords and punctuation marks. Then, a stemming step was applied to the remaining words. This step is required in order to map each word into its root or stem [19]. The tf-idf technique was used to transform the pre-processed text into a sparse vector representation. To compute the importance of a word *w*, the technique considers the internal frequency of *w* in a single document. Moreover, the internal frequency is compared with the relative frequency of *w* in all documents of the dataset [20]. The tf-idf representation of *w* in a document *d* is computed as

$$\begin{array}{c} \text{tf-idf}\left(w,d\right)=\frac{f(w,d)}{n_{d}}.\frac{\text{log}\;N}{\text{log}(N_{w})}, \end{array}$$ (1)

where *f*(*w*, *d*) represents the frequency *w* in *d*, *n*_*d*_ is the number of words in *d*, *N* stands for the total number of documents in the dataset, and *N*_*w*_ represents the number of documents in which *w* appears at least once.

We used the tf-idf vector representations of each abstract as input values of a K-Means based method for short texts KE. We also used the tf-idf weight of each word from the full content of the papers in order to give an importance value for a long text KE method (LKE).

### 3.3 Network creation

Owing to the limited length of paper abstracts, the extraction of statistically significant information from individual texts is unfeasible. In the case of concise textual content, the creation of intricate network structures and the acquisition of pertinent word frequency data are unattainable. Consequently, we have employed network representation techniques to derive additional information with the objective of improving the keyword extraction process.

Two different network models are used in our study. In order to cluster *short texts* into groups of related papers, we used a citation network for the *short texts* keyword extraction task. In this case, the network structure represents the whole dataset of documents. Conversely, when extracting keywords from long texts, each text is modeled as a word co-occurrence network [21–23].

In short text keyword extraction, citation networks are employed to depict the relationships among papers, based on the assumption that papers connected through citations are inherently related. After obtaining this network, clusters of papers within it are identified. Subsequently, these paper clusters are leveraged using an approach akin to the tf-idf technique. Words that exhibit higher significance within the cluster in comparison to their occurrence outside the cluster are regarded as the most representative for that particular cluster. After identifying the most representative words for the cluster, the paper-level keywords are those that appear in the abstract of each respective paper.

The unweighted paper citation network was built following the methodology described in [7]. The citation networks are intended to represent a semantical similarity structure that do not use textual information to establish links between papers. The resulting network was composed of 11, 063 nodes and 94, 472 edges. The community structure of this network and the information of title and abstract are then used to detect the most important words in each network community.

The *full content* of a paper is modeled as a word co-occurrence network. In this graph model, each node represents a word, and the edges between two nodes are based on the neighborhood relationship of two words. We used the approach that can include virtual links, so that similar words can be linked. This model and its variations have been used in many different scenarios [24–27]. The networks were characterized using well-known centrality measurements to rank the words according to their structural importance in the networks [28]. The main idea behind this technique is that the most central nodes in the network also correspond to the most central words in the text.

### 3.4 Community detection

This phase is responsible for detecting communities, i.e. clusters of papers linked via citation links. Communities are groups of nodes that are more densely interconnected with each other in comparison with the rest of the nodes from the network [29]. The identification of communities in large networks is quite a useful task. For example, the nodes that belong to the same community likely share several common properties. Also, the number of found communities and their respective features could help to identify the category of a network for classification tasks [30]. The identification of communities is also useful to understand the dynamic evolution and organization of a network [31]. In this paper, we evaluated the following methods: Multilevel, Label Propagation, Infomap, Fast Greedy, and Leiden method [32–36]. In the S1 Appendix, we provide a brief description of each method.

In the context of community detection methods, we investigated if community-based methods are consistent in the sense that they generate well-defined, large communities. This is an important step in our analysis because small communities can lead to low performance [7]. In the paper citation network encompassing approximately 11,000 nodes, the majority of community detection methods yielded results with a range of 23 to 39 paper communities. These communities typically consist of more than 100 papers. Notably, the application of the infomap algorithm resulted in the identification of over 400 distinct communities, the majority of which were characterized by fewer than 10 papers. Consequently, we made the decision to disregard communities with a limited number of papers prior to computing the relevance of individual words.

### 3.5 Short texts keyword extraction

This step consists of the extraction of keywords from the pre-processed paper abstracts. We evaluated a network community-based approach that generates a word importance index to rank each word from the paper abstracts. For comparison purposes, we also evaluated tf-idf and K-Means-based methods for the short texts KE task. Both techniques are well-suited for the task as they are standard statistical methods employed in the analysis of document collections within a corpus. The tf-idf approach involves comparing a document to all other documents in the dataset. In contrast, the KMeans approach involves comparing the cluster under examination to all other clusters within the document.

1. *Community-based approach:* we used the community structure found from papers citation networks to detect the word importance index of each word from paper abstracts. Words exhibiting a higher prevalence within a cluster of papers than outside it indicate that they appear more frequently in the specific context of the cluster than in a broader context, outside of it. To put it differently, words that are more frequently encountered within the cluster are, in turn, more likely to be associated with the topic represented by that cluster, as opposed to more general terms that could occur in any document. Consequently, the contrast in relative frequency plays a pivotal role in discerning words characterized by distinctiveness and intrinsic importance within the defined cluster.
  The adopted index quantifies the relative frequency of a word appearing inside a community against its frequency in the remaining documents of the citation network [7]. To compute the word importance index *I* for a word *w*, we first compute the frequency of the word inside a community *α*. This quantity is the relative internal frequency $F_{\alpha }^{\left(\text{in}\right)}\left(w\right)$, given by
  $$\begin{array}{c} F_{\alpha }^{\left(\text{in}\right)}\left(w\right)=\frac{n_{\alpha }\left(w\right)}{\left|\alpha \right|}, \end{array}$$ (2)
  where *n*_*α*_(*w*) is the total number of papers containing *w* appears within a community *α*, and |*α*| represents the number of papers associated with a community *α*. We also compute the relative frequency of *w* outside *α*, $F_{\alpha }^{\left(\text{out}\right)}\left(w\right)$, which is computed as:
  $$\begin{array}{c} F_{\alpha }^{\left(\text{out}\right)}\left(w\right)=\sum_{\gamma \neq \alpha }\frac{n_{\gamma }\left(w\right)}{N-|\alpha |}, \end{array}$$ (3)
  where *N* is the total number of papers in the network. Then, the importance index *I*(*w*) is calculated as the highest difference between the relative in-community and out-community frequencies, i.e.:
  $$\begin{array}{c} I\left(w\right)=\underset{\alpha }{\text{max}}[F_{\alpha }^{\left(\text{in}\right)}\left(w\right)-F_{\alpha }^{\left(\text{out}\right)}\left(w\right)]. \end{array}$$ (4)
  The word importance index was computed for all words from paper abstracts, and then the best-ranked words were considered as relevant keywords for each abstract.
2. *tf-idf based approach:* the tf-idf values considering all paper abstracts from the dataset are computed. For each abstract, we considered the tf-idf weights of the words comprising the abstract. The words with the highest tf-idf values were selected as relevant keywords for each abstract.
3. *K-Means based approach:* this method is equivalent to the *community-based approach*. The difference is that clusters are obtained via the K-Means algorithm [37]. To obtain the cluster, we first obtained the embedding of each abstract. Then we evaluated several values of *K* to find the optimal number of clusters.

### 3.6 Long texts keyword extraction

The keywords obtained from full texts are considered reference keywords when evaluating the quality of keywords extracted from short texts. Full-text content encapsulates the entirety of information within a paper, thereby presumptively offering a comprehensive representation of the author’s intended content as opposed to the abstract. Because an established set of ideal keywords (referred to as gold standard keywords) is unavailable, we choose the part of the manuscript with the largest informational content—the full text—on the basis that it is more likely to contain keywords of higher informativeness.

Here we considered as input texts the complete content of the research papers. We adopted several methods found in the literature to extract keywords documents. The methods can be classified into two approaches: statistical and graph-based approaches:

- *Statistical and traditional keyword extraction methods:* In this step we employed statistical techniques that are commonly used for keyword extraction tasks. These methods perform an appropriate analysis of the statistical distribution of words along documents. The main goal of statistical methods is to detect and rank relevant words of documents without any *a priori* or external information [8]. The methods we adopted are based on frequency, word tf-idf, word entropy, word intermittency, and Yake. We also evaluated a graph-based approach named TextRank, and a method that uses word embeddings based on BERT. The methods are described in the S1 Appendix.
- *Network-based methods:* a comprehensive set of centrality measures were used to analyze the word co-occurrence networks derived from the full content of the papers. Co-occurrence networks serve as simplified syntactical representations of texts. This implies that words occupying central syntactical positions tend to hold greater significance within the text, as observed by many works [38, 39]. The network measurements are useful to identify the most relevant nodes in a network [28]. Therefore, they allow ranking the nodes according to their topological importance so that they can find the most important words for each text [39] by finding the most important nodes of the network. We selected as keywords for each text the best-ranked nodes (words) according to the following centrality metrics: degree, PageRank, betweenness, eigenvector centrality, closeness and accessibility computed at the first two levels [40]. We also employed a methodology that combines the results of each centrality metric. In the methodology henceforth referred to as voting system, the keywords found by the majority of the network measurements were selected as relevant keywords for each text.

## 4. Results and discussion

Our analysis is divided into two sections. Section 4.1 describes a statistical analysis of the datasets. Section 4.1 provides a comparison of keywords extracted from short and full-text sources. We also analyze the performance of distinct network community methods for the task.

### 4.1 Dataset analysis and selection of reference keywords

In this section, we first perform a statistical evaluation of the datasets through the analysis of the number of common words between the paper abstracts (short-size texts) and the full content of the research paper (long-size texts). This analysis is an initial step to the generation of a set of gold standard keywords for each paper abstract. Because many datasets comprising full-text papers lack keywords selected by human experts, we used as a starting point the full content (including all sections except the abstract) of each paper. We employed keyword extraction methods to extract reference keywords from the complete content of each paper. However, we first analyzed the number of mutual words existing between each abstract and the full content. In some cases, it is possible that the paper authors use specific words to express their main ideas in the abstract and they could change to other words using synonyms or similar expressions in the rest of the paper. Therefore, it becomes important to analyze whether the information extracted from full texts is compatible with the content of abstracts.

**Fig 2:**
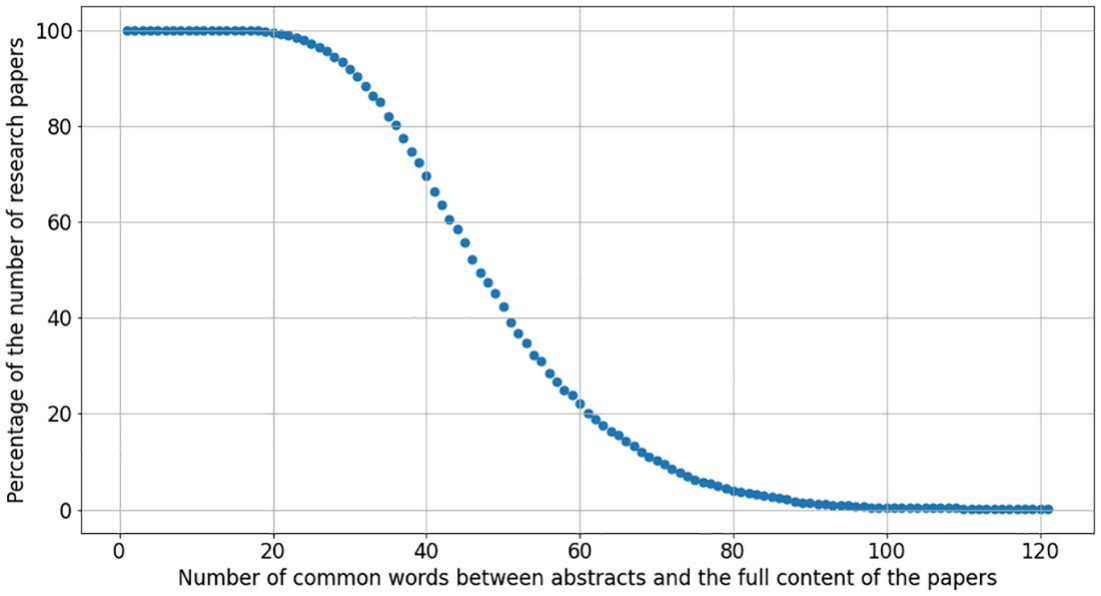
Analysis of the shared word count between research paper abstracts and their full-text contents. The x-axis denotes the number of overlapping words between paper abstracts and their respective full texts. The y-axis represents the probability *P*(*x* ≥ *w*), indicating the proportion of papers that contain at least *w* shared words between their abstracts and full texts.

We computed how many words (*w*) in the abstract are also present in the full content of the papers. The cumulative distribution (i.e. *P*(*x* ≥ *w*)) of this quantity in the dataset is shown in Fig 2. A significant number of research papers (80%) present a high number of common words (40) between the abstracts and the full content of the papers. 50% of the papers have at least 50 common words. As expected, this means that most of the information in the abstract is also available in the remainder of the paper.

**Fig 3:**
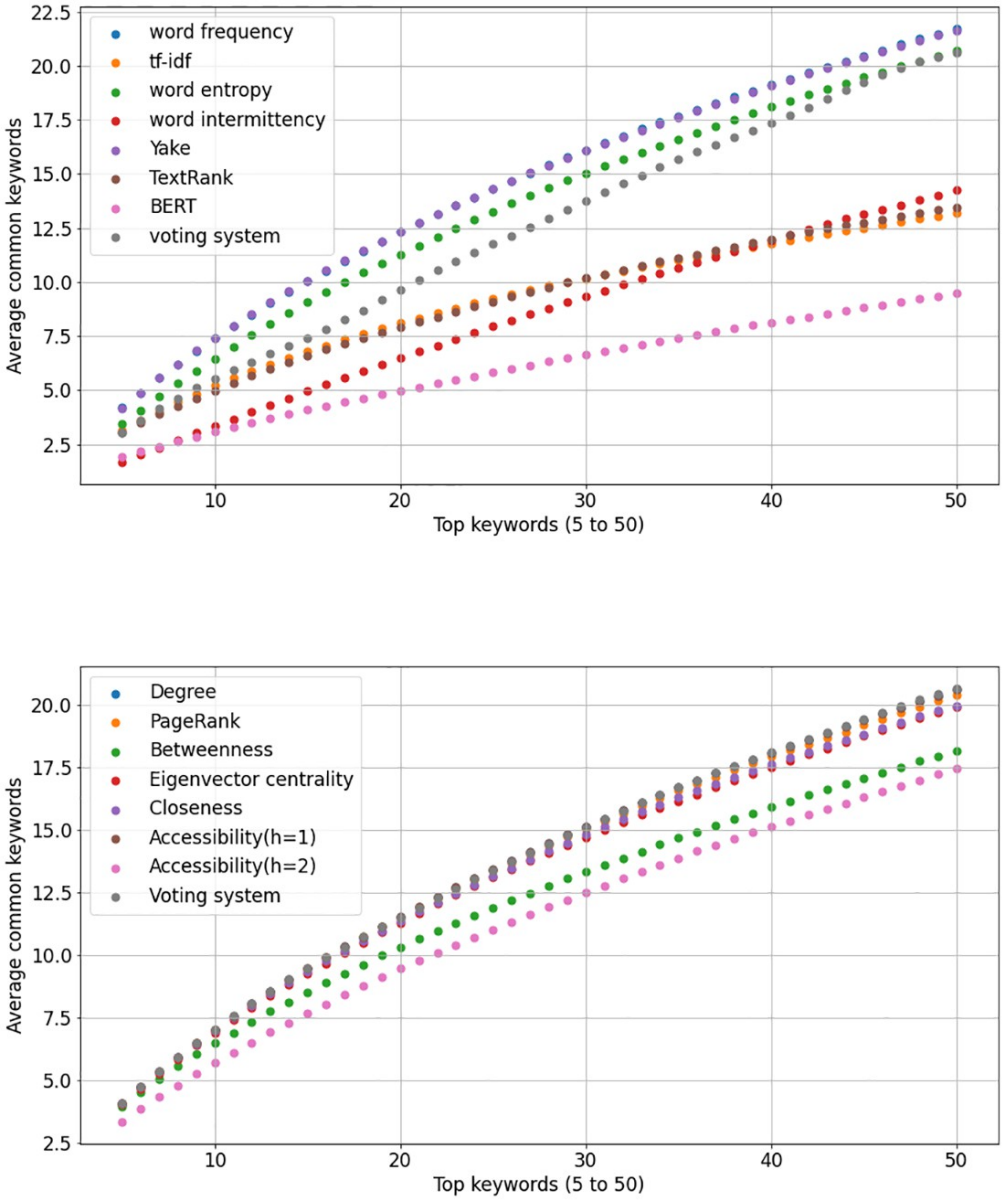
Analysis of the overlap between the common words found in the paper abstracts and those identified by keyword extraction methods for longer texts (i.e., the full content of the papers). The x-axis depicts the count of keywords retrieved from the full content, and the y-axis illustrates the average number of retrieved keywords that concurrently appear in the paper abstract. The upper figure illustrates the analysis conducted via traditional and statistical keyword extraction methods applied to the full paper dataset. In contrast, the lower figure portrays the analysis conducted via network-based keyword extraction methods.

Now we evaluate how many *keywords* found in the *full content analysis* are also present in the abstract. We used two approaches to extract reference keywords considering each paper’s full content: statistical and graph-based KE methods. We evaluated these approaches by counting the number of mutual words between the keywords generated by each method and the words composing the paper abstracts. Fig 3 depicts the obtained results for each approach. According to the size of the abstracts and the full content (see Table 1), we considered recovering between 5 and 50 keywords generated by each KE method. Then, we count the number of these keywords that are part of the abstracts.

In relation to traditional and statistical methods, the results displayed in the upper panel of Fig 3 show that the methods Yake, word frequency, and word entropy outperformed the other KE techniques. These methods were able to find the largest number of common words with the abstracts. The voting system approach did not achieve the best results. The methods based on word intermittency and BERT also displayed a low number of mutual words with the paper abstracts. We also evaluated the methods based on word co-occurrence networks and centrality measurements. The results depicted in the bottom panel of Fig 3 suggest that almost all centrality metrics performed similarly. We observed that the voting system, node degree, PageRank, and accessibility (computed at the first hierarchical level) outperformed the other network-based methods. However, the difference in terms of performance with the other network metrics is not significant.

### 4.2 Extracting keywords from abstracts

In this section, we analyze if the methods adopted to extract *keywords from abstracts* are able to capture keywords that are found when the full-text content is analyzed. We used accuracy as a performance evaluation measure. The performance of the methods is measured in terms of the number of common words between the reference keywords and the keywords generated by the short-text KE methods, divided by the total number of reference keywords. We established a parameter *N* to represent the number of reference keywords to be considered in the evaluation. As reference keywords (i.e. keywords obtained from full texts), we used the methods with the highest performance observed in the previous section.

**Fig 4:**
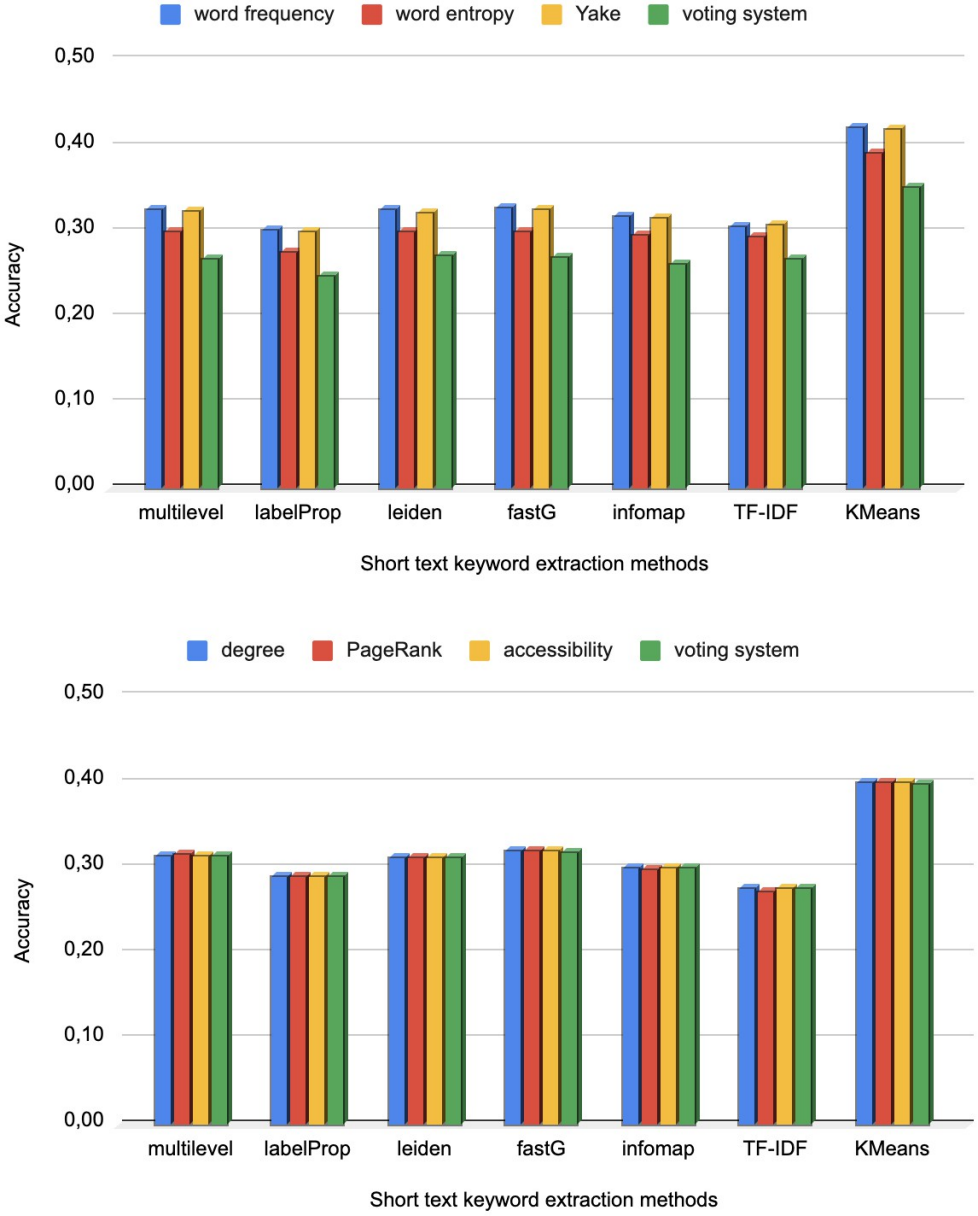
Comparative analysis of the accuracy derived from the evaluation of keyword extraction methods for short texts (paper abstracts). We extracted a total of *N* = 30 keywords from full texts. The upper figure illustrates the performance analysis when traditional and statistical methods are employed to extract reference keywords. The lower figure portrays the accuracy analysis with network-based methods as the benchmark for reference keywords.

The results in the upper panel of Fig 4 show the performance analysis considering as reference keywords the ones obtained from statistical methods. The results suggest that community-based approaches obtained similar accuracy values since no method clearly outperformed the others. The label propagation method achieved a slightly lower performance than the other methods. We also found that the tf-idf method displayed a performance that is similar to the other network community-based methods. Surprisingly, when citation information is disregarded and only the textual information is used, the performance is improved. The K-Means method is significantly better than all other approaches, with a gain of 25% in performance, in some cases. The complete analysis considering different values for the parameter *N* is shown in the S1 Appendix.

The bottom panel of Fig 4 shows the performance analysis considering as reference keywords the ones obtained from (co-occurrence) network-based methods. Here we see that all considered network centrality metrics provide almost the same performance. This result is consistent with the literature in text network analysis since there is a correlation of centrality metrics when analyzing written texts. Concerning the formation of reference keywords via community detection methods, it is also worth noting that all community-based methods provided similar performance for the task. Conversely, choosing reference keywords via tf-idf yielded the worst performance. Once again, the best performance was obtained with the KMeans method.

One possible explanation for the similar performance achieved by the community-based detection methods could be the fact that all methods are generating similar partitions and, consequently, they are selecting the same set of keywords for each paper abstract. In order to evaluate this hypothesis, we computed the Spearman correlation coefficient of the ranking of words’ relevance generated by different community detection methods. The results (not shown) revealed that the methods are actually selecting different sets of keywords. If one considers the full rank of words, the Spearman correlations are typically lower than 0.10. In a similar fashion, when considering only the top 30 ranked words, all correlations were below 0.27. As expected, in both scenarios, the highest correlation was found for the rankings generated by the Multilevel and Leiden methods [32, 34].

The performance results revealed interesting insights. First, we found that identifying keywords from citation information alone does not provide the highest match between keywords found in abstract and full-text. While citations have been used in numerous contexts [41], one possible reason for the observed low performance is that citations may not reflect the semantic similarity of texts, which may hinder the performance of the community detection methods. In fact, some studies have pointed out a discrepancy between citation and content similarity. For example, [41] found that citation and content similarity are not consistent since the most similar papers are oftentimes disregarded when selecting references for papers. In a similar fashion, the differences in the content have been used to improve models reproducing the growth of citation networks, since content similarity has also been used as an important feature to model the growth of citation networks [42].

While the use of textual information was able to provide a better performance in recovering keywords from full-text content, the obtained accuracy is still below 50%. This means that using cluster information from papers abstracts is not enough to recover the full content of papers. This may have implications in many studies that are based on recovering text content based on keywords. For example, when studying the properties of citation networks, the selection of papers via keywords may affect the stability of citation network metrics. A different number of communities depicting subfields of a major area can be found if the keywords terms are not well-defined to select the relevant papers.

The differences in content extracted from abstracts and full texts can also potentially lead to distinct interpretations in the context of *Science of Science*. In a document similarity network, for example, the centrality of a paper may strongly depend on the use of abstracts or full texts. If such networks are studied in other contexts, this may lead to less robust conclusions. For example, comparing the semantic similarity between papers linked by citations may lead to different results depending on how much text is used to gauge semantic similarity [41]. Therefore, it remains relevant to consider full-text content to draw conclusions relying upon the analysis of papers’ semantic similarity.

## 5. Conclusion

The identification of keywords from short texts poses a significant challenge. In this paper, we evaluated whether well-known approaches are able to extract keywords from abstracts that are compatible with a full-content analysis. Due to the limited context provided in abstracts, we employed methods that leverage the citation context to cluster papers into semantically similar groups. Additionally, we used strategies based on statistics and the K-Means algorithm. Reference keywords were obtained from the full content of papers through the use of multiple techniques.

In our initial research question, we assessed whether clustering abstracts via community detection on citations could enhance the quality of the extracted keywords. We initially observed comparable performance across the various community detection methods, with no distinct superiority demonstrated by any specific method. However, the most significant insight emerged when we found that a straightforward approach, namely the K-Means algorithm, outperformed methods based on communities derived from citation networks. The findings indicate that a simple clustering approach may be more efficient than methods that rely on communities derived from citation networks.

All in all, citation networks and alternative methods that do not rely on citations demonstrated suboptimal performance. This result implies that the keywords obtained from abstracts are not consistent with those obtained from a comprehensive content analysis. Consequently, further research is necessary to investigate whether the observed variations may lead to discrepancies in the analysis of document similarity networks [43]. This could prove to be a pivotal research question in future studies, as varying conclusions may arise when handling results that hinge on the source of the paper from which the information is derived. One pertinent example pertains to the analysis of fields’ evolution, as these fields can be contingent on the specific definition of keywords.

One way to potentially enhance the performance of clustering methods for keyword extraction is through the use of alternative methods for text vector representation. The incorporation of text embeddings, such as those generated by the BERT model [44], may assist in effectively representing the documents. Additionally, implementing synonym handling during the generation of reference keywords could also prove beneficial. The performance could also be improved by integrating citation and text-based information when creating paper networks.

## Supporting information

S1 Appendix (PDF)

S1 Data (ZIP)
