# Supplementary material for: Using citation networks to evaluate the impact of text length on keyword extraction
Source: PLoS One. 2023 Nov 27;18(11):e0294500. doi: 10.1371/journal.pone.0294500 (PMC10681196; doi:10.1371/journal.pone.0294500)
Supplement: S1 Appendix — (PDF) [file pone.0294500.s001.pdf]

Appendix

Community detection methods

In this section, we provide a brief description of the network community methods employed in this paper:

1. *Multilevel*: in this algorithm, each node is assigned to a different community. Then nodes are moved to the communities of their corresponding neighbors that yield the highest positive contribution to modularity [1]. This process is repeated until the local contribution of nodes to the modularity is no longer improved. Each community from the original network is reduced into a single node (maintaining the total weight of the adjacent edges) and the method continues to the next level. The algorithm ends when there is no longer any possibility of increasing the modularity score after reducing communities to nodes.

2. *Label propagation:* The method presented in [2] is based on the principles of neighborhood connectivity and information diffusion in networks. The approach begins by assigning unique community labels to each node in the network. These labels are subsequently propagated throughout the network. During each iteration, each node adopts the most prevalent label within its immediate neighborhood. The edges within the network are then randomly removed, and the nodes are updated in a random order before the next iteration commences. The algorithm stops when the nodes reach a consensus, which is defined as a state in which each node holds the majority label among its neighboring nodes.

3. *Leiden:* The Leiden algorithm, which was proposed in [3], represents an improvement to the widely-used multilevel method [1]. The latter is known to have a weakness of often discovering communities that are weakly connected. In contrast, the Leiden method aims at ensuring that communities are well-connected through the implementation of the following three phases: (i) local moving of nodes (as in the multilevel method); (ii) refinement of partitions; and (iii) aggregation of the network. By incorporating these three phases, the Leiden algorithm is able to uncover higher-quality clusters in significantly less time when compared to the multilevel method.

4. *Fast Greedy:* this algorithm is based on hierarchical agglomerative clustering and aims to optimize the modularity score [4]. The method begins by considering a subnetwork composed exclusively of edges between highly-connected nodes. This methodology subsequently evaluates randomly selected edges that improve the modularity of the subnetwork and aggregates them. This process is repeated until the incremental improvement in modularity becomes negligible. Finally, the communities are obtained by identifying the connected components within the subnetwork.

5. *Infomap:* the algorithm was introduced in [5] and is based on information theory. This method begins by encoding the network into modules in a manner that maximizes the amount of information retained from the original network. The encoded network is then transmitted through a channel with limited capacity. The goal of the decoder is to attempt to decode the message and construct a set of possible candidates for the original network. The fewer the number of candidates, the more information about the original network has been transmitted. The algorithm also uses random walks to analyze the flow of information through the network.

Statistical keyword detection

- *Word frequency and tf-idf methods (Freq. and tf-idf):* one of the simplest techniques for keyword extraction is the frequency-based approach, which assigns relevance to words that occur at a high frequency. The words that rank the highest in terms of frequency are therefore considered as keywords. In order to mitigate the limitations of the frequency-based methods, we also evaluated the tf-idf method. Unlike the frequency-based approach, the tf-idf method assigns a weight to the frequency of each word based on its number of occurrences within the document as well as throughout the entire dataset. In this approach, the words with the highest tf-idf values are considered as keywords.

- *Word entropy (W.E.):* This method leverages Shannon's entropy to analyze the information content of the sequence of occurrences of each word in a given text [6]. This technique requires partitioning the texts into N segments to calculate the entropy of each word. In this study, we partitioned the paper texts according to the number of sentences that make up each text. According to this method, the higher the value of entropy of a word, the greater the heterogeneity of the distribution of that word within the text, and thus the greater its relevance. One of the key advantages of this method is that it does not require a large text corpus for training; it only requires the input text.

- *Word intermittency (W.I.):* This metric takes into account the relationship between the significance of a word and its spatial distribution [7]. Previous research has found that important words are closely related to the main topics of the text and display a highly heterogeneous distribution. Such words tend to be located in specific regions of the text, exhibit large frequency fluctuations and often form clusters [6]. In contrast, common words such as stopwords are distributed randomly throughout the document and exhibit a relatively homogeneous distribution. Thus, as proposed in [6], a statistical analysis of the distribution of word occurrences can be employed to identify relevant keywords within a given text. Similar to the frequency and entropy methods, this technique identifies important words solely based on the target text and does not require external information.

- *Yake*: The Yake method extracts statistical features from the source text to identify the most relevant keywords [8]. Five features are computed for each individual term: (i) casing, (ii) word positional, which assigns greater importance to words that appear at the beginning of a text, (iii) word frequency, which assigns relevance to words that occur more frequently, (iv) word relatedness to context, which measures the number of different terms that appear to the left and right of the target word, and (v) word *difSentence*, which measures how often a word appears across different sentences. These features are then combined into a single measure to assign an importance weight to each word. According to this metric, terms exhibiting lower scores are identified as keywords [9].

- *TextRank (TextR):* The TextRank method, proposed in [10], is a graph-based approach that employs the PageRank algorithm and is widely used for text summarization and keyword extraction tasks. In this method, texts are modeled as word co-occurrence networks, where the nodes are represented by words and edges are established between two nodes if they co-occur within a window size. In the original paper, the window size was set between 2 and 10 words. The PageRank algorithm is employed to rank each word, and the top-ranked words are selected as relevant keywords.

- *BERT-based method*: the Bidirectional Encoder Representations from Transformers (BERT) technique is a state-of-the-art embedding model that captures the semantic content of documents through dense vector representations [11]. The BERT-based method generates word embeddings for each n-gram in the text. Subsequently, the cosine similarity metric is applied to identify the words that are most similar to the original document. The top-ranking similar words are then considered as relevant keywords for each document.

- *Voting system (V.S.):* To enhance the precision of the long text keyword extraction methods, we integrated the outcomes of the aforementioned approaches. We employed a consensus-based technique, wherein the keywords identified by the majority of extraction methods were selected.

Complete results based on accuracy analysis

The accuracy of keyword extraction methods for short texts (paper abstracts) is presented in Tables 2 and 3. In Table 2, the results are based on keywords identified using traditional and statistical methods applied to the entire content of the papers. In contrast, the results in Table 3 are derived from the most significant words identified by network-based methods applied to the full paper content.

Table 2. Accuracy obtained from the evaluation of keyword extraction methods for short texts (paper abstracts). Here we considered as reference keywords the relevant words found by the *traditional and statistical* methods for the full content of the papers. N represents the number of top keywords we recovered for both short and long texts keyword extraction methods.

	**Word Frequency**				**tf-idf**			
**method**	**N=10**	**N=20**	**N=30**	**N=40**	**N=10**	**N=20**	**N=30**	**N=40**
multilevel	0.1578	0.2559	0.3269	0.3678	0.0740	0.1066	0.1448	0.1793
labelProp	0.1375	0.2312	0.3022	0.3504	0.0787	0.1033	0.1363	0.1724
leiden	0.1496	0.2533	0.3260	0.3664	0.0819	0.1153	0.1463	0.1785
fastG	0.1465	0.2518	0.3290	0.3684	0.0713	0.1023	0.1419	0.1767
infomap	0.1375	0.2320	0.3175	0.3647	0.1458	0.1561	0.1726	0.1951
tf-idf	0.2858	0.2889	0.3071	0.3350	0.3596	0.3223	0.2984	0.2755
KMeans	0.4253	0.4213	0.4220	0.4146	0.2129	0.2106	0.2198	0.2279
	**Word Entropy**				**Word Intermittency**			
**method**	**N=10**	**N=20**	**N=30**	**N=40**	**N=10**	**N=20**	**N=30**	**N=40**
multilevel	0.1341	0.2294	0.3004	0.3460	0.0679	0.1291	0.1828	0.2233
labelProp	0.1165	0.2052	0.2769	0.3285	0.0608	0.1159	0.1663	0.2110
leiden	0.1279	0.2293	0.3007	0.3455	0.0651	0.1288	0.1829	0.2228
fastG	0.1237	0.2232	0.3012	0.3456	0.0658	0.1241	0.1807	0.2218
infomap	0.1259	0.2166	0.2967	0.3441	0.0715	0.1274	0.1811	0.2225
tf-idf	0.2477	0.2708	0.2945	0.3217	0.1093	0.1539	0.1868	0.2180
KMeans	0.3596	0.3835	0.3929	0.3925	0.1335	0.1921	0.2280	0.2499
	**Yake**				**TextRank**			
**method**	**N=10**	**N=20**	**N=30**	**N=40**	**N=10**	**N=20**	**N=30**	**N=40**
multilevel	0.1553	0.2536	0.3242	0.3650	0.1138	0.1689	0.2101	0.2326
labelProp	0.1362	0.2290	0.2997	0.3481	0.0967	0.1594	0.2012	0.2260
leiden	0.1509	0.2516	0.3226	0.3633	0.0986	0.1673	0.2090	0.2319
fastG	0.1448	0.2485	0.3256	0.3653	0.1172	0.1818	0.2184	0.2356
infomap	0.1400	0.2309	0.3153	0.3616	0.0829	0.1503	0.2034	0.2302
tf-idf	0.2905	0.2937	0.3091	0.3353	0.1381	0.1492	0.1703	0.1951
KMeans	0.4221	0.4190	0.4194	0.4121	0.2132	0.2337	0.2459	0.2479
	**BERT**				**Voting System**			
**method**	**N=10**	**N=20**	**N=30**	**N=40**	**N=10**	**N=20**	**N=30**	**N=40**
multilevel	0.0573	0.0924	0.1254	0.1501	0.1231	0.1925	0.2688	0.3283
labelProp	0.0710	0.0936	0.1221	0.1462	0.1108	0.1758	0.2485	0.3123
leiden	0.0757	0.1092	0.1325	0.1518	0.1325	0.2003	0.2716	0.3274
fastG	0.0739	0.0965	0.1257	0.1494	0.1300	0.1941	0.2702	0.3280
infomap	0.0596	0.0938	0.1261	0.1483	0.1115	0.1826	0.2633	0.3251
tf-idf	0.0899	0.1043	0.1207	0.1394	0.1891	0.2280	0.2687	0.3070
KMeans	0.1804	0.1668	0.1688	0.1726	0.2761	0.3110	0.3531	0.3746

Table 3. Accuracy obtained from the evaluation of keyword extraction methods for short texts (paper abstracts). Here we considered as reference keywords the most important words found by the *network-based* methods for the full content of the papers. N is the number of top keywords we recovered for both short and long texts keyword extraction methods.

	**Degree**				**PageRank**			
**method**	**N=10**	**N=20**	**N=30**	**N=40**	**N=10**	**N=20**	**N=30**	**N=40**
multilevel	0.1605	0.2490	0.3149	0.3539	0.1622	0.2498	0.3153	0.3514
labelProp	0.1324	0.2234	0.2903	0.3374	0.1329	0.2247	0.2899	0.3349
leiden	0.1417	0.2421	0.3125	0.3522	0.1428	0.2429	0.3120	0.3494
fastG	0.1466	0.2496	0.3195	0.3549	0.1481	0.2510	0.3194	0.3523
infomap	0.1256	0.2152	0.3002	0.3476	0.1247	0.2146	0.2979	0.3445
tf-idf	0.2529	0.2546	0.2757	0.3085	0.2516	0.2521	0.2725	0.3043
KMeans	0.4047	0.3989	0.4010	0.3948	0.4070	0.3998	0.3999	0.3920
	**Betweenness**				**Eigenvector**			
**method**	**N=10**	**N=20**	**N=30**	**N=40**	**N=10**	**N=20**	**N=30**	**N=40**
multilevel	0.1563	0.2281	0.2809	0.3127	0.1484	0.2385	0.3008	0.3385
labelProp	0.1255	0.2021	0.2571	0.2973	0.1253	0.2136	0.2787	0.3238
leiden	0.1356	0.2202	0.2774	0.3099	0.1343	0.2315	0.2994	0.3370
fastG	0.1392	0.2272	0.2836	0.3134	0.1387	0.2373	0.3031	0.3393
infomap	0.1125	0.1855	0.2596	0.3028	0.1239	0.2120	0.2921	0.3346
tf-idf	0.2291	0.2255	0.2387	0.2661	0.2490	0.2522	0.2726	0.3021
KMeans	0.3828	0.3656	0.3574	0.3503	0.3865	0.3844	0.3847	0.3797
	**Closeness**				**Accessibility (h=1)**			
**method**	**N=10**	**N=20**	**N=30**	**N=40**	**N=10**	**N=20**	**N=30**	**N=40**
multilevel	0.1585	0.2438	0.3066	0.3426	0.1605	0.2490	0.3149	0.3539
labelProp	0.1317	0.2198	0.2855	0.3278	0.1324	0.2234	0.2903	0.3374
leiden	0.1390	0.2373	0.3052	0.3411	0.1417	0.2421	0.3125	0.3522
fastG	0.1461	0.2446	0.3096	0.3431	0.1466	0.2496	0.3195	0.3549
infomap	0.1209	0.2127	0.2932	0.3367	0.1256	0.2152	0.3002	0.3476
tf-idf	0.2478	0.2499	0.2717	0.3019	0.2529	0.2546	0.2757	0.3085
KMeans	0.3936	0.3883	0.3891	0.3828	0.4047	0.3989	0.4010	0.3948
	**Accessibility(h=2)**				**Voting System**			
**method**	**N=10**	**N=20**	**N=30**	**N=40**	**N=10**	**N=20**	**N=30**	**N=40**
multilevel	0.1196	0.1943	0.2526	0.2904	0.1605	0.2481	0.3141	0.3535
labelProp	0.1048	0.1744	0.2338	0.2777	0.1324	0.2226	0.2909	0.3377
leiden	0.1088	0.1869	0.2508	0.2894	0.1426	0.2408	0.3119	0.3522
fastG	0.1108	0.1909	0.2519	0.2899	0.1464	0.2487	0.3175	0.3548
infomap	0.0997	0.1740	0.2419	0.2855	0.1255	0.2147	0.3001	0.3479
tf-idf	0.1977	0.2125	0.2344	0.2638	0.2536	0.2546	0.2759	0.3093
KMeans	0.3162	0.3167	0.3236	0.3264	0.4039	0.3963	0.3985	0.3952

References

1. Blondel VD, Guillaume JL, Lambiotte R, Lefebvre E. Fast unfolding of communities in large networks. Journal of Statistical Mechanics: Theory and Experiment. 2008;2008(10):P10008. doi:10.1088/1742-5468/2008/10/p10008.

2. Raghavan UN, Albert R, Kumara S. Near linear time algorithm to detect community structures in large-scale networks. Phys Rev E. 2007;76:036106. doi:10.1103/PhysRevE.76.036106.

3. Traag VA, Waltman L, Van Eck NJ. From Louvain to Leiden: guaranteeing well-connected communities. Scientific reports. 2019;9(1):1–12.

4. Clauset A, Newman ME, Moore C. Finding community structure in very large networks. Physical review E. 2004;70(6):066111.

5. Rosvall M, Bergstrom CT. Maps of random walks on complex networks reveal community structure. Proceedings of the national academy of sciences. 2008;105(4):1118–1123.

6. Carretero-Campos C, Bernaola-Galván P, Coronado A, Carpena P. Improving statistical keyword detection in short texts: Entropic and clustering approaches. Physica A: Statistical Mechanics and its Applications. 2013;392(6):1481–1492.

7. Amancio DR. Probing the topological properties of complex networks modeling short written texts. PloS one. 2015;10(2):e0118394.

8. Campos R, Mangaravite V, Pasquali A, Jorge A, Nunes C, Jatowt A. YAKE! Keyword extraction from single documents using multiple local features. Information Sciences. 2020;509:257–289.

9. Campos R, Mangaravite V, Pasquali A, Jorge AM, Nunes C, Jatowt A. Yake! collection-independent automatic keyword extractor. In: European Conference on Information Retrieval. Springer; 2018. p. 806–810.

10. Mihalcea R, Tarau P. Textrank: Bringing order into text. In: Proceedings of the 2004 conference on empirical methods in natural language processing; 2004. p. 404–411.

11. Devlin J, Chang MW, Lee K, Toutanova K. Bert: Pre-training of deep bidirectional transformers for language understanding. arXiv preprint arXiv:181004805. 2018;.
